# Analysis of the types of social activity participation of older adults living in the community and the health level and quality of life by types: Applying Latent Class Analysis

**DOI:** 10.1002/alz.094765

**Published:** 2025-01-09

**Authors:** sang Geun Kim, Yunyeong Jeong, chae Yeon Hwang, Kang‐Hyun Park

**Affiliations:** ^1^ Baekseok University, Cheonan‐si Korea, Republic of (South); ^2^ Baekseok University, Cheonan‐si, Dongnam‐gu Korea, Republic of (South)

## Abstract

**Background:**

Participation in various social activities of elderly has a significant impact on overall health and quality of life, including physical and metal health. Therefore, in this study, we aim to analyze the types of social activity participation among community‐dwelling elderly and examine differences in characteristics, health status, and quality of life according to these types of participation of social activities. Thus, we purpose to utilize the findings of this study as a foundation basis for research aimed at promoting services and intervention related to social activities of elderly in community.

**Method:**

This study utilized the 9th wave (2022) panel data from the Korean Longitudinal Study of Elderly Employment(KLoSA). Latent class analysis was utilized to explore types of social activities. To assess the goodness of fit of the latent model, χ² (Chi‐square) test, Akaike Information Criteria (AIC), Bayesian Information Criteria (BIC), Sample‐Size Adjusted Bayesian Information Criteria (SABIC) indices, and Lo‐Mendell‐Rubin adjusted Likelihood Ratio Test (LMR‐LRT) were employed. One‐way ANOVA was conducted to examine characteristics across different types.

**Result:**

The study included a total of 6,057 elderly individuals aged 60 and above residing in the local community for analysis. The results demonstrate four distinct types of social activity participation among the elderly. These four groups were named based on the types of social activities they engaged in: “Religious and Social Activity Centered,” “Social and Leisure Activity Centered,” “Social Activity Centered,” and “Inactive.” The analysis of characteristics across these types showed statistically significant differences in demographic factors such as age, marital status, and religion. In terms of health‐related aspects, subjective health and overall quality of life indices exhibited statistically significant differences among the types.

**Conclusion:**

Social activities among the elderly were analyzed into four distinct types based on the activities they engaged in. The participation of elderly individuals in various social activities is considered a crucial indicator for improving health and quality of life. It is hoped that this study will serve as a foundational basis for developing services and intervention programs for elderly individuals in the community, aimed at facilitating their participation in diverse social activities.

